# Large Vestibular Schwannoma: A Two-Stage Surgery

**DOI:** 10.7759/cureus.33552

**Published:** 2023-01-09

**Authors:** José Orlando de Melo Junior, José Alberto Landeiro, Roberto Leal da Silveira

**Affiliations:** 1 Neurosurgery Department, Paulo Niemeyer State Brain Institute, Rio de Janeiro, BRA; 2 Neurosurgery Department, Antonio Pedro University Hospital, Rio de Janeiro, BRA; 3 Neurosurgery Department, Madre Teresa Hospital, Belo Horizonte, BRA

**Keywords:** cerebellopontine angle tumour, acoustic neuroma, vestibular schwannoma, staged surgery, retrosigmoid approach

## Abstract

Treatment of large vestibular schwannoma (VS) has historically centered on total resection of the lesion. Staged surgery has been used for VS that is highly vascularized, unexpected events during surgery, and thinned and stretched facial nerve with serious adherence causing difficult dissection. We present a case of a patient with a large VS resected through a two-stage surgery through the same retrosigmoid craniotomy.

## Introduction

Treatment of large vestibular schwannoma (VS) has historically centered on total resection of the lesion. This approach has been linked to a significant rate of functional impairment of the facial and cochlear nerves [1-3]⁠. There is no established agreement for small- and medium-sized lesions; however, surgery remains the best choice for large lesions [4]⁠. We present a case of a patient with a large VS resected through a two-stage surgery achieving total resection with an acceptable facial nerve function outcome.

## Case presentation

**Figure 1:**
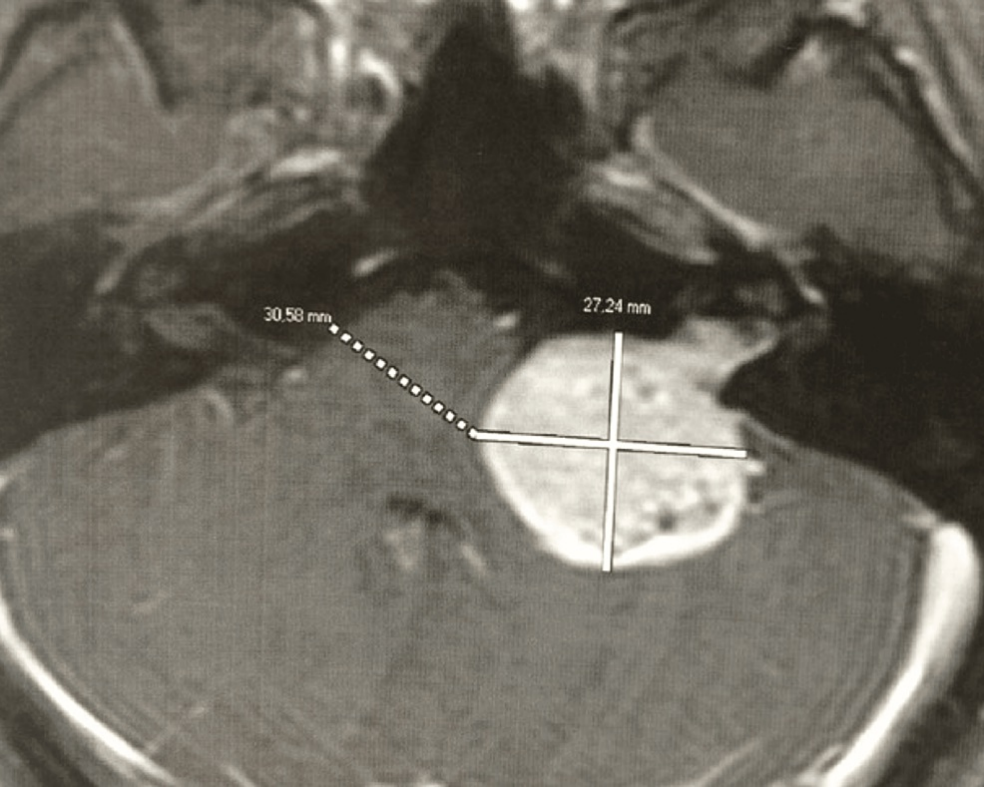
Axial contrast-enhanced T1-weighted MRI showed a large left-sided VS (two crossed white lines), grade T4b (Hannover classification), with severe brainstem compression

A 27-year-old woman was getting ready to get married in 2017. She set the wedding date and sent out invitations to everyone. A month before the date of the celebration, she tried on the dress and the special heel but was unable to stand and walk properly due to the intense feeling of imbalance and instability. Neurological examination showed left ear hearing loss and cerebellar ataxia. The MRI revealed a large left vestibular schwannoma (VS), grade T4b (Hannover classification), with severe compression of the brainstem (Figure 1). There was a long conversation and discussion about the possible treatments, and she opted for surgical treatment in hopes of improving symptoms. However, she did not accept the result of facial paralysis, mainly because it was going to stay forever in the photos of the wedding party with her face paralyzed. Therefore, we proposed a partial removal through a retrosigmoid approach in an attempt to save the facial nerve function and to improve the imbalance and instability, and a second stage some months after the wedding celebration for complete removal, perhaps with the risks of facial nerve palsy. At that time, she accepted the surgery in two stages, and thus, everything was done based on this free consent waiver contract.

Description of the technique

**Figure 2:**
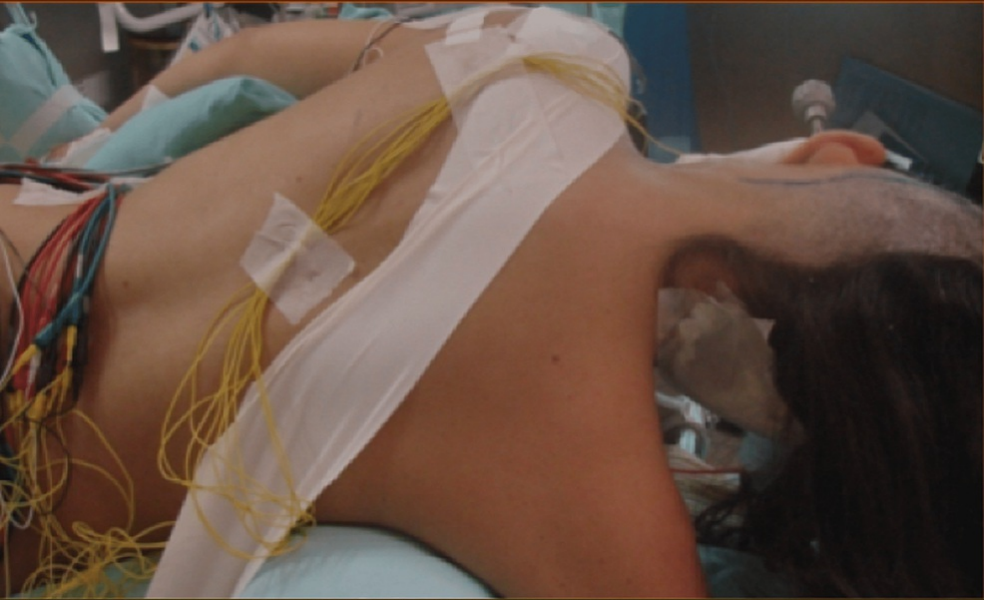
Lateral decubitus position. The patient’s head was positioned parallel to the floor, flexed, and slightly tilted to the contralateral side

The patient was placed in a lateral decubitus position (Figure 2). Intraoperative electrophysiological monitoring was applied with somatosensory and motor-evoked potentials, auditory-evoked potentials, and direct facial nerve stimulation.

**Figure 3:**
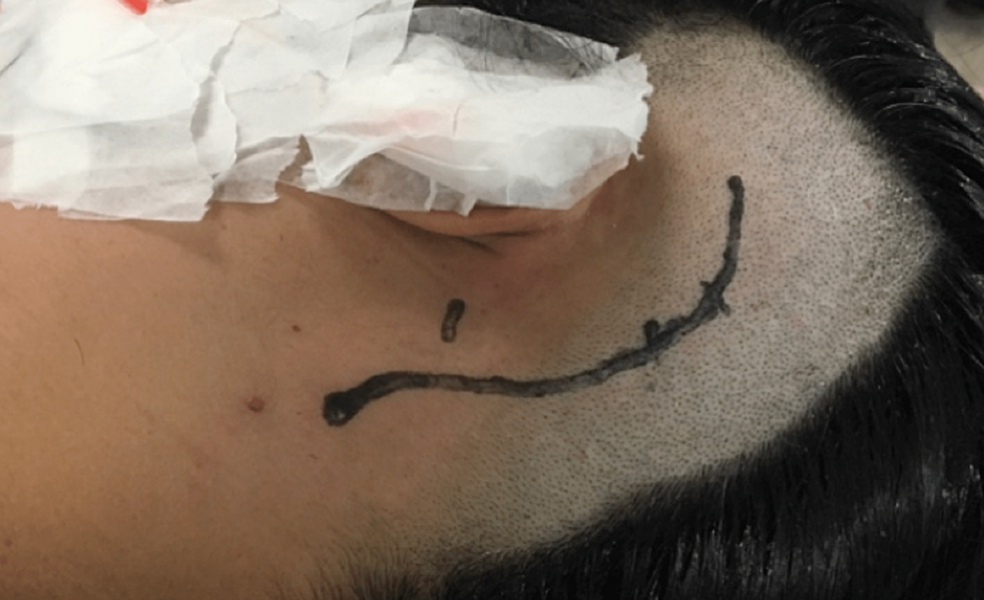
Curvilinear skin incision

**Figure 4:**
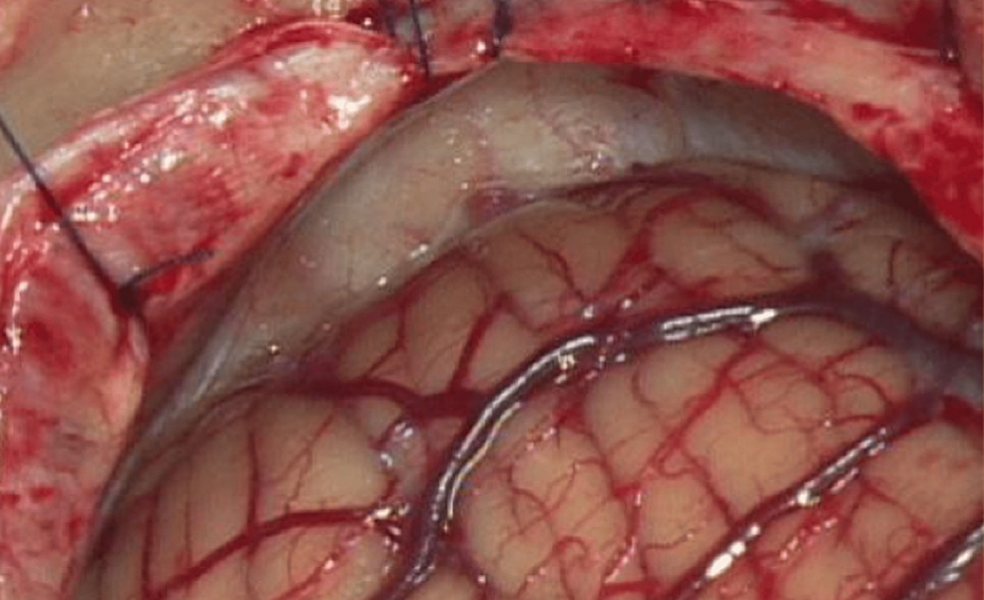
Retrosigmoid craniotomy

A curved skin incision was placed two fingers behind the ear (Figure 3). The subcutaneous and muscle planes were incised in line with the skin incision. A burr-hole was placed in the asterion, and a retrosigmoid craniotomy was carried out (Figure 4). The mastoid air cells were closed with bone wax to prevent CSF fistula. Dura was opened, in a semicircular fashion, with its base dressing the sigmoid sinus.

**Figure 5:**
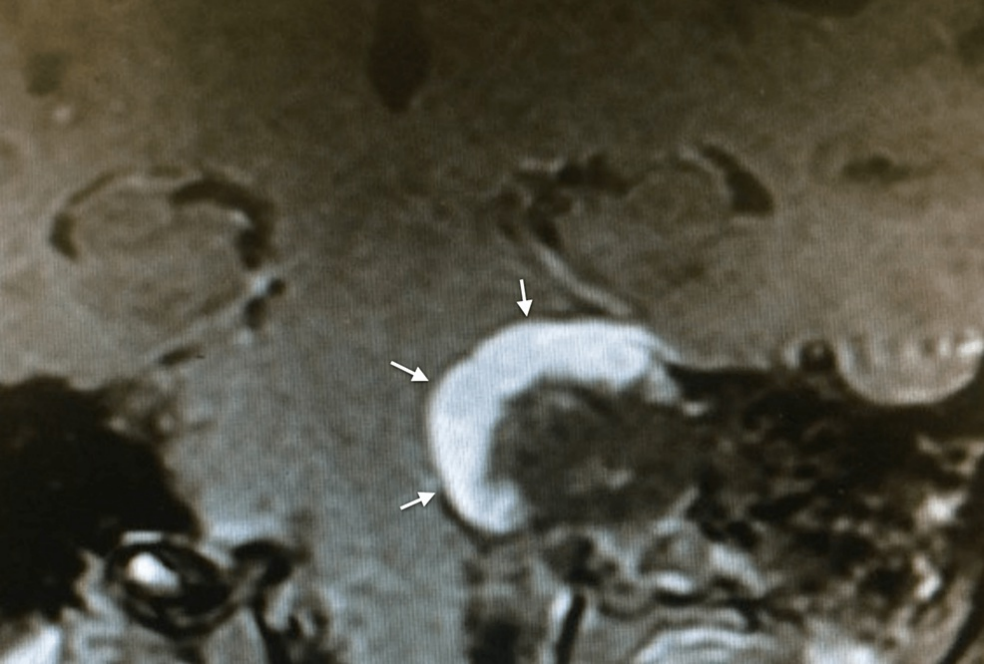
Postoperative coronal contrast-enhanced T1-weighted MRI showed subtotal resection (white arrows)

Cerebrospinal fluid was drained through the cerebellomedullary cistern for cerebellar relaxation. The cerebellum was gently retracted with a spatula to expose the CPA. The tumor capsule was opened, and the tumor was removed with an ultrasonic aspirator. The arachnoid plane was kept intact to protect the neurovascular structures. The posterior lip of the internal auditory was drilled, and the tumor could be removed meticulously using a micro dissector and microhooks. In the first surgery, the tumor was removed partially. Resection was stopped as soon as the facial motor evoked potential began to decrease. The dura was sutured in a watertight fashion. The bone flap was fixed with titanium mini plates and screws. The wound was closed in a watertight fashion. The patient improved her symptoms of imbalance and instability without facial nerve palsy, House-Brackmann (HB) grade I, and was able to get married. Postoperative MRI showed subtotal resection (Figure 5).

**Figure 6:**
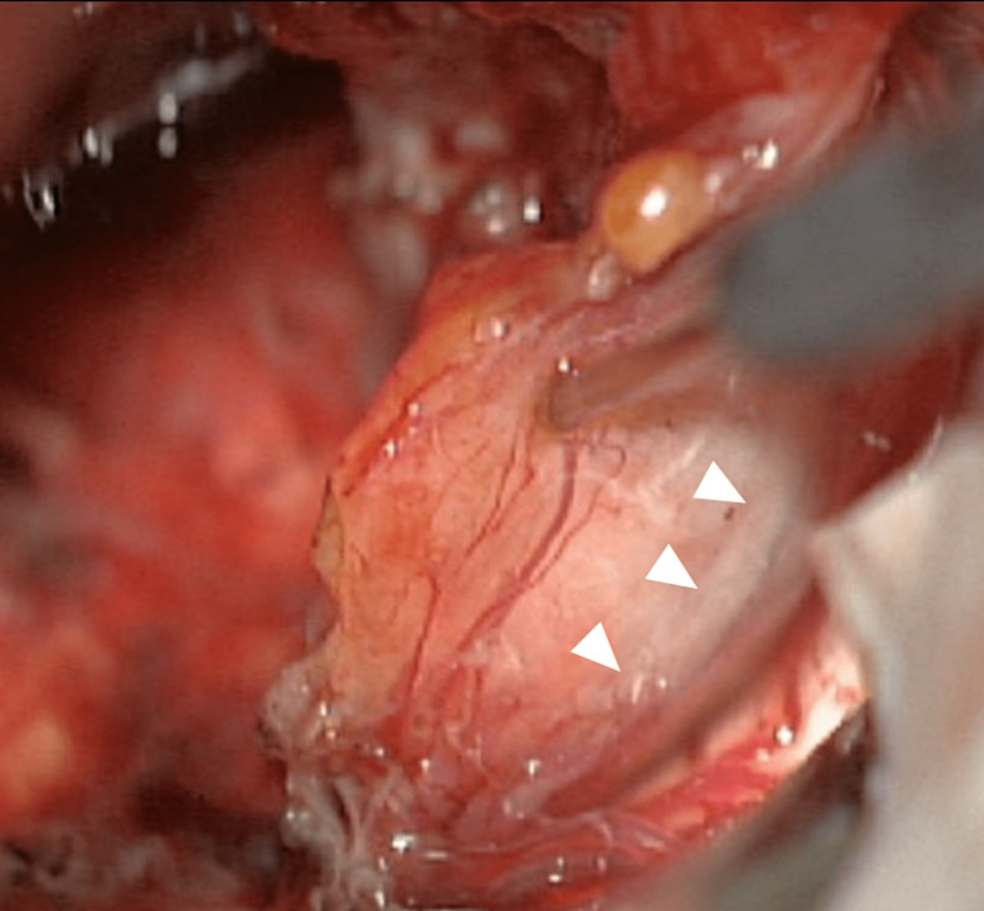
The facial nerve thinned and spread out over the capsule (white arrowheads)

**Figure 7:**
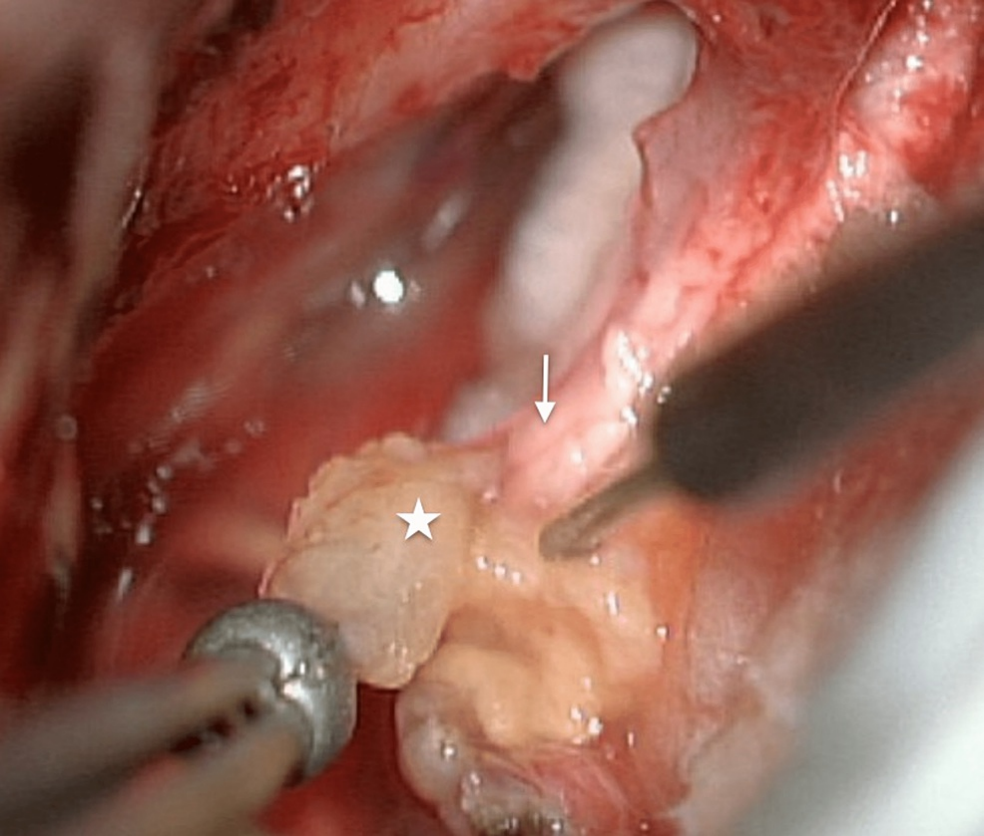
The facial nerve (white arrow) and last piece of residual tumor (star)

**Figure 8:**
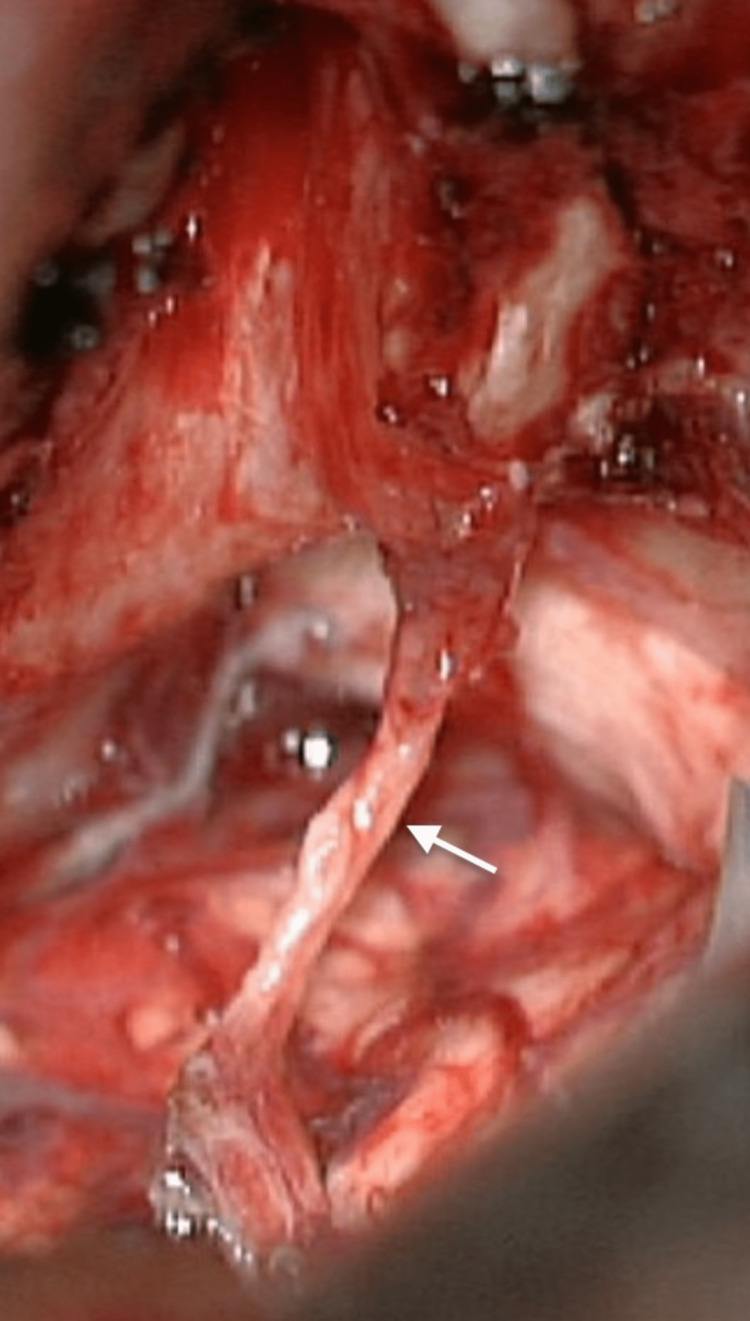
Total resection with anatomically preserved facial nerve (white arrow)

**Figure 9:**
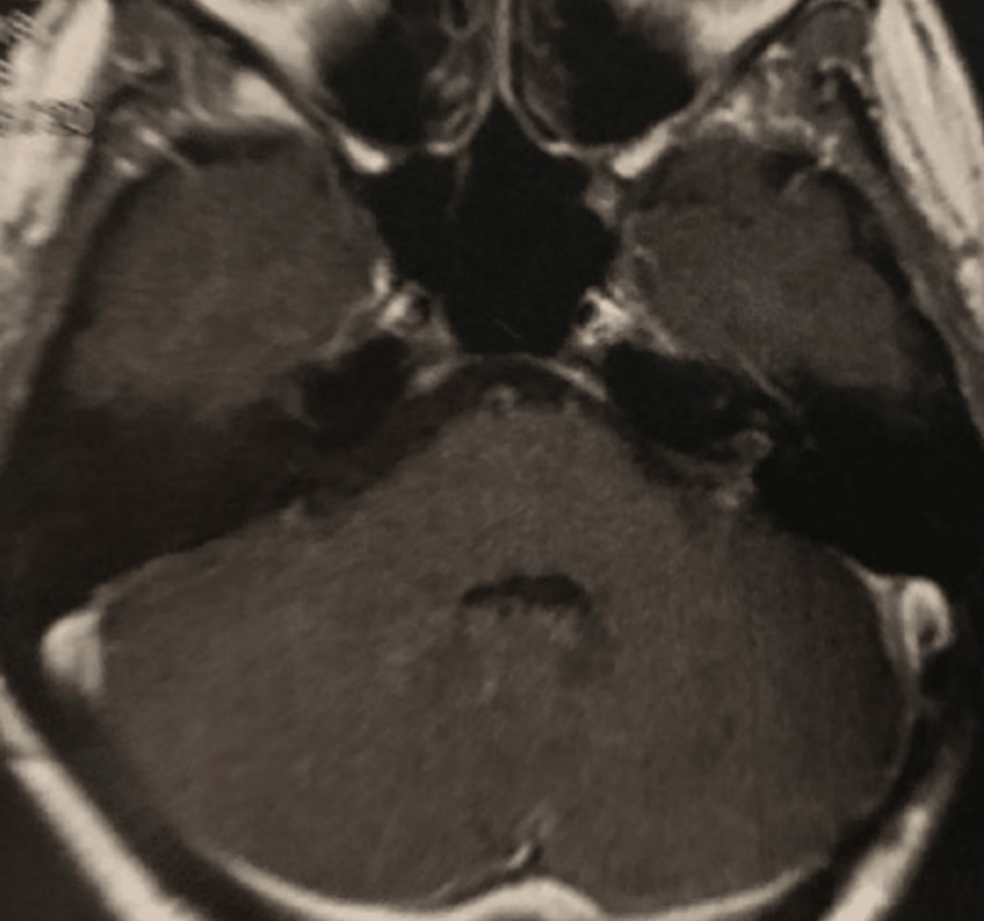
Postoperative axial contrast-enhanced T1-weighted MRI showed total resection

**Figure 10:**
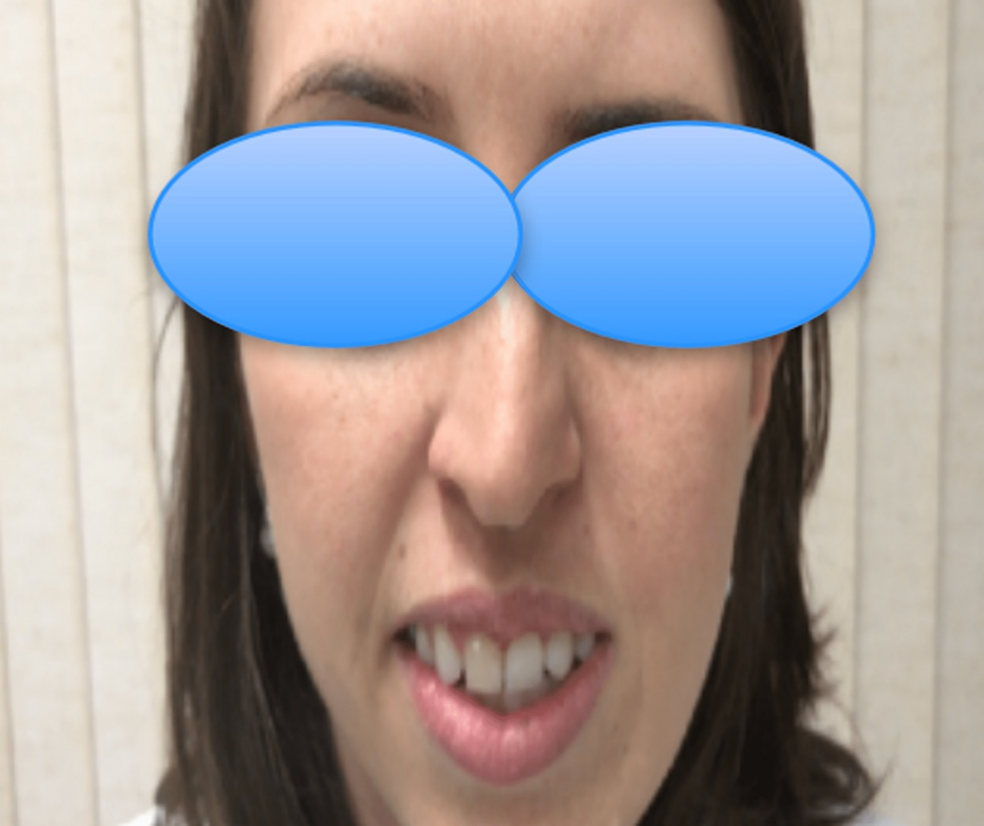
Good recovery of facial nerve function at follow-up

The second stage was performed six months later using the same retrosigmoid approach. The scar was extensively released to provide adequate exposure to the residual tumor. The facial nerve was thinned and spread out over the capsule (Figures 6, 7). A total resection was achieved (Figure 8). Postoperative MRI showed total resection (Figure 9). The patient had a facial nerve palsy HB grade IV after the second surgery, which improved at the follow-up to HB grade II (Figure 10). Hearing status decreased after surgery.

## Discussion

Treatment of large vestibular schwannoma (VS) has historically centered on total resection of the lesion. This approach has been linked to a significant rate of functional impairment of the facial and cochlear nerves [1-3]⁠. There is no established agreement for small and medium-sized lesions; however, surgery remains the best choice for large lesions [4]⁠.

During the past decades, the focus has been progressively changing from total to subtotal resection to preserve the facial nerve function and patients’ quality of life [5,6]⁠. However, Nakatomi et al. have shown that tumor recurrence was predicted by the degree of resection, with subtotal resection having almost 11-fold greater risk compared with gross total resection [7]⁠. Therefore, subtotal resection carries the risk of long-term recurrence.

Staged resection for large VS has been indicated as an option to optimize facial nerve outcomes and reduce complications [8,9]⁠. Two-stage surgery using translabyrinthine-translabyrinthine, retrosigmoid-translabyrinthine, and retrosigmoid-retrosigmoid approaches have been reported to address these lesions [8-12]. The stage surgery may be used for the following situations: highly vascularized VS with severe brainstem compression, unexpected events during surgery like as cerebellar swelling or vital sign changes, thinned and stretched facial nerve with serious adherence causing difficult dissection, decreased facial nerve potential, and the existence of brainstem/cerebellum edema, are the main reasons for staged resection [8-11,13,14]⁠.

## Conclusions

Staged resection for large VS has been considered an exception to optimize facial nerve outcomes and reduce complications. Although there remain no absolute indications, it may be considered an option for selected cases.
